# The variable use of heparin through intravenous bolus and flush fluid systems during endovascular stroke treatment, a world-wide survey

**DOI:** 10.1186/s42155-025-00532-3

**Published:** 2025-03-03

**Authors:** Senta Frol, Faysal Benali, Aymeric Rouchaud, Robrecht R. M. M. Knapen, Wim H. van Zwam, Research Committee of the European Society of Minimally Invasive Neurological Therapy (ESMINT)

**Affiliations:** 1 Department of Vascular Neurology, University Medical Center Ljubljana, Ljubljana, Slovenia; 2 Faculty of Medicine, University of Ljubljana, Ljubljana, Slovenia; 3 Department of Radiology, AZ Vesalius, Tongeren, Belgium; 4 Department of Radiology and Nuclear Medicine, Maastricht University Medical Center+ and CARIM, School for Cardiovascular Diseases, Maastricht University, Maastricht, the Netherlands; 5 Department of Interventional Neuroradiology, Univ Limoges, XLIM, CNRS Umr7252. Limoges University Hospital, Limoges, France

**Keywords:** Heparin, Endovascular treatment, Stroke

## Abstract

**Background:**

The total amount of heparin administered through flush fluids in stroke patients is not considered in recent trials, possibly influencing main results. We investigated the use of heparin among treating physicians worldwide.

**Methods:**

We conducted a survey from November 2022 to January 2023 to identify the variability of heparin administration during stroke endovascular treatment (EVT). We calculated the total heparin dose per hour (IU/h) by adding the intravenous (IV)-bolus dose to the amount administered through flush fluids, calculated by a multiplication of the number of infusion bags, drip rate[mL/h] and heparin concentration[IU/L].

**Results:**

A total of 315 participants from different countries worldwide completed the survey and 231/315(73%) respondents administer heparin during EVT. The majority administered heparin only through flush fluids (168/231; 72.7%), followed by both IV-bolus and flush fluids (36/231; 16%), and those who used only an IV-bolus (27/231; 11.7%). From the participants that administer heparin through flush fluids, the median heparin concentration was 2000 IU/L (range:100 IU/L-10000 IU/L). The total heparin dose (administered through flush fluids and IV-bolus) among 23 respondents showed a median of 4650 IU/h (IQR:3432–5900). Among the respondents who administer heparin through IV-bolus only, the median was 5250 IU (IQR:3750–7500).

**Conclusion:**

This survey revealed variable heparin doses administered by physicians worldwide during EVT and reflects the lack of international guidelines. Caution is warranted, specifically during complex/long EVT procedures. Furthermore, heparin flush doses should be considered in future trials regarding periprocedural anticoagulants, since imbalances could potentially confound results.

**Supplementary Information:**

The online version contains supplementary material available at 10.1186/s42155-025-00532-3.

## Introduction

Endovascular treatment (EVT) is the standard of care for patients with acute ischemic stroke (AIS) due to large vessel occlusion (LVO). Systemic unfractionated heparin is commonly used during EVT to continuously flush catheters and guidewires, preventing clot formation at catheter tips.

Despite its benefits, heparin's strong anticoagulant effect poses significant risks. Prior analyses by a Netherlands research group have shown that increasing heparin flush fluid doses is associated with a higher risk of symptomatic intracranial haemorrhage (sICH) without reducing the risk of distal embolization or improving three-month functional outcomes [1, 2]. A higher risk of sICH with heparin during EVT was also reported in the multicentre randomized clinical trial of endovascular treatment for acute ischemic stroke in the Netherlands (MR CLEAN-MED) [3]. Notably, this large randomized trial did not account for the additional heparin dose administered through flush fluids. This omission raises the possibility that adjusting for this form of heparin administration could significantly alter the study's outcomes.

Furthermore, there is a lack of literature providing guidelines on the optimal or maximal total dose of heparin that should be administered through flush fluids during EVT, leading to substantial variability in practice among treating physicians.

The aim of this international survey is to gain insight into current practices regarding heparin use during EVT among treating physicians worldwide.

## Methods

In collaboration with the European Society of Minimally Invasive Neurological Therapy (ESMINT), we conducted a survey among EVT treating physicians from countries across Europe, Asia, and Canada. All ESMINT members were requested to complete the survey online (see Supplemental File 1). The survey was open and available for 3 months (from November 2022 to January 2023).

The survey was divided into three main sections addressing: (i) administration of heparin through IV bolus alone, (ii) administration through flush fluids alone, and (iii) administration through both IV bolus and flush fluids. Each section included specific questions regarding the concentration of heparin in the saline flush fluid (IU/L), the dose of the IV bolus (IU/kg), and the drip rate of the heparinized flush fluid (mL/h) (Supplemental File 1).

Respondents were asked whether the drip rate was managed automatically (perfusor) or manually (drip chamber from an infusion bag). For those managing it manually, we recorded standard drip rates (36 mL/h, 90 mL/h, 180 mL/h, and 900 mL/h) and presented these rates to respondents through corresponding online videos [4].

Additionally, data were collected on the number of infusion bags used, the timing of connecting the infusion bag, and the circumstances under which interventionists decided to withhold the administration of heparin.

The total dose of heparin entering the patient through flush fluids per hour was calculated using the following formula: (Number of infusion bags) x (Drip rate [mL/h]) x (Heparin concentration [IU/L]) x (1/1000 L/mL). When different drip rates were used for different lines, the individual drip rates were added to calculate the total infusion rate per hour.

## Results

Out of 4005 invited participants, 315 responded to the survey. Among the participants, 231 (73%) administered heparin (Table 1).

**Table 1:** Participants and heparin characteristics from the survey

	Participants *N* = 315
Type of responders – n (%)	
(Neuro-)radiologists	211 (67)
Neurologist	37 (11,8)
Neurosurgeon	30 (9,5)
Not specified	77 (11,8)
Male sex – n (%)	255 (81)
Level of experience – n (%)	
< 1 year	15 (4,8)
1–5 years	99 (31,4)
5–10 years	80 (25,4)
> 10 years	121 (38,4)
Heparin usage – n (%)	
Yes	231 (73)
No	84 (27)
Heparin administration – n (%) *N* = 231	
Flush fluid	168 (73)
IV Bolus	27 (12)
Flush + IV Bolus	36 (16)
Access heparin administration – n (%)	
Femoral	115 (49,8)
Carotid	13 (5,6)
Radial	39 (16,9)
Brachial	11 (4,8)
Not specified / all of the above^a^	95 (41,1)

^a^Responders could select different options for this question, therefore the total number exceeds 231

### Number of infusion bags / lines

The majority of respondents who administered heparinized flush fluids used three infusion bags (75/148; 50.7%), while 12/148 (8.1%) used four infusion bags during the procedure.

### Preferred drip rate for flush fluids heparin

**Fig. 1:**
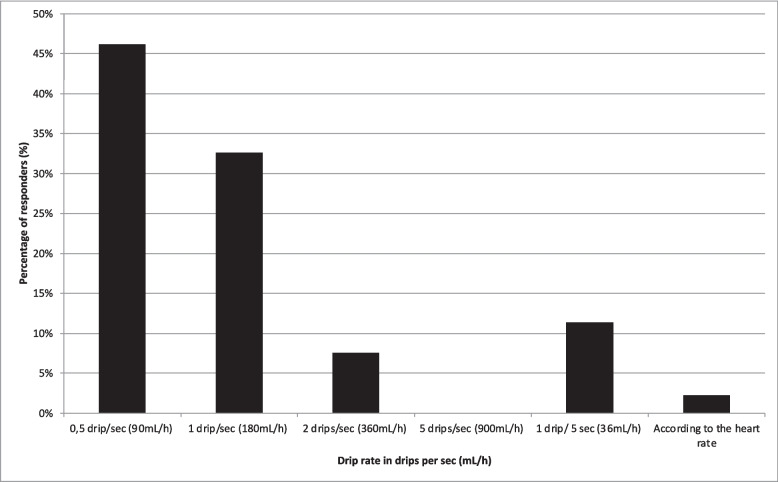
Preferred drip rate for automatic perfusors (*n* = 132)

Among the participants, 168 treating physicians administer heparinized flush fluid alone and 36 in combination with an IV bolus. Of the 204 responders that use heparin in flush fluids, 148 (72.5%) answered the question on how and how much they set their preferred drip rate. The majority (135/148; 91.2%) set their drip rate manually. As to the question of what drip rate the interventionists preferred when they set their drip rate automatically 132 answered, of which 61 (46%) indicated a rate of 0.5 drips per second (i.e., 90 mL/h); 43 (32.6%) indicated a rate of 1 drip per second (i.e. 180 mL/h); 10 (7.6%) indicated a rate of 2 drips per second (i.e. 360 mL/h); 15 (11.4%) indicated a rate of 1 drip per 5 s (i.e. 36 mL/h) and 3 (2.3%) indicated that they state their rate according to the heart rate (Fig. 1).

### Timing of connecting the infusion bag

One hundred forty-two (69.9%) participants responded to the question on when to connect the infusion bag and 26 (18%) reported connecting the infusion bag when the carotid artery was catheterized, while the remaining 82% connected the bags just before or after groin puncture.

### Dose of heparin flush fluids

Out of 204 responders that use heparin in flush fluids, 169 provided data on heparin concentration (IU/L). The median heparin concentration was 2000 IU/L (IQR 1000–5000 IU/L), and the concentrations ranged from a minimum of 100 IU/L to a maximum of 10,000 IU/L.

### IV bolus – dose

Among the 231 participants who administer heparin, 27 reported administering it through an IV bolus. Of these, 21 provided data on the dose. The median IV heparin dose was 5250 IU (IQR 3750–7500 IU). Doses from 37.5 IU to 75,000 IU were reported. Notably, this maximum dose was administered only as an IV bolus and not in addition to heparinized flush fluid. Furthermore, we assume reporting mistakes in the extremes. Additionally, 93% (25 out of 27) of the responses indicated that the bolus is given immediately after groin puncture.

### Total calculated heparin dose per hour through heparinized flush fluids

**Fig. 2:**
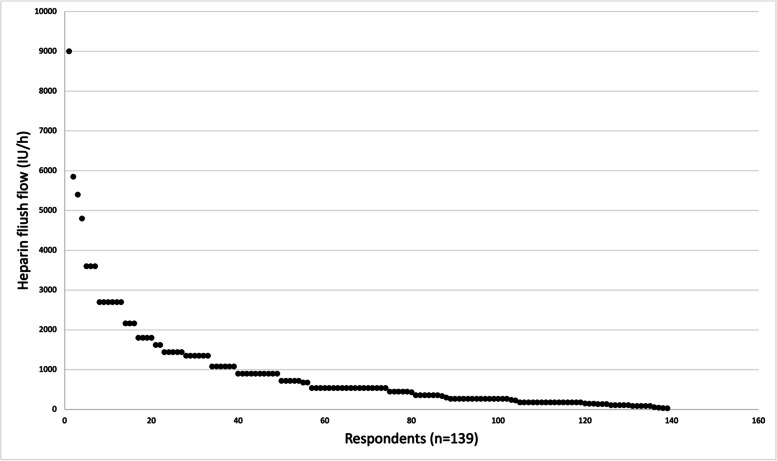
Scatterplot of calculated total heparin dose per hour through flush fluids

We were able to calculate the total heparin dose administered through flush fluids per hour for 139 respondents. This total dose ranged from 27 IU/h to 9000 IU/h. The median dose was 540 IU/h (IQR: 180–1080 IU/h) (see Table S1 and Fig. 2).

### Total calculated heparin dose per hour through IV bolus + heparinized flush fluids

**Fig. 3:**
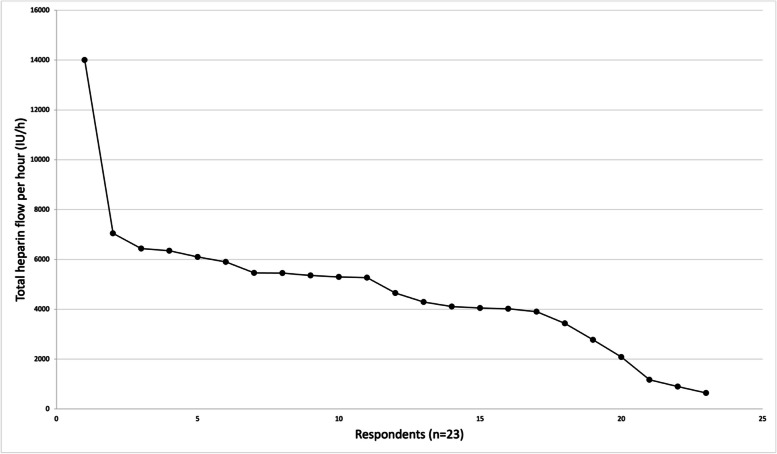
Scatterplot of calculated total heparin dose per hour for administered IV bolus

For those who administer heparin through both flush fluids and IV bolus, we were able to calculate the total hourly heparin dose in 23 respondents (see Table S2 and Fig. 3). If respondents provided their heparin dose (IU) per kg bodyweight, we re-calculated this dose for an average body weight of 75 kg. The total heparin dose ranged from 640 IU/h to 14,000 IU/h, with a median of 4650 IU/h (IQR 3432–5900 IU/h).

### Withholding heparin

When participants were asked about circumstances under which heparin administration was withheld, the majority reported that heparin in flush fluids was omitted when IV thrombolysis had already been administered (40/69 respondents; 58%) or when the Alberta Stroke Program Early CT Score (ASPECTS) at baseline was less than 5 (24/69 respondents; 35%). Other reasons for withholding heparin included a history of heparin-induced thrombocytopenia (HIT), anticipation of straightforward complete recanalization, multiple femoral punctures, concurrent use of direct oral anticoagulants or warfarin, and planned extracranial or intracranial stenting.

## Discussion

In this paper, we present the findings of an international survey on the use of heparin in saline flush infusion bags and/or as intravenous (IV) bolus during EVT in patients with AIS due to LVO.

The use of heparin as a flush fluid or bolus during EVT varies widely across different countries and even within regions: some interventionalists use heparin routinely, while others avoid it, indicating a lack of consensus on the use of heparin administration during EVT [2, 5]. Current guidelines regarding the periprocedural use of heparin do not provide specific recommendations, and the use is most often at the discretion of the treating physician [6]. Although heparin is used in nearly all interventions, differences in administration, concentration, and routine use between treating physicians and hospitals are reported among various studies [7–9]. A review done by Wiersema et al. draws attention toward large variations in the use of heparin during peripheral arterial interventions and advocates for the mandatory assessment of actual anticoagulation status [10].

Balancing the benefits of reducing thrombotic complications with the potential harm of heparin remains crucial, particularly in AIS, where the infarcted brain tissue is highly susceptible to haemorrhagic transformation. Heparin can increase the risk of bleeding during and after EVT, including the development of symptomatic intracranial haemorrhage (sICH) [3, 11]. Additionally, heparin-induced thrombocytopenia (HIT) is a rare but potential serious immune-mediated adverse reaction [12].

Literature evaluating the impact of heparin on the outcomes of EVT shows ambiguous results. The MR CLEAN-MED study assessed the safety and efficacy of administering IV aspirin (300 mg), unfractionated heparin (5000 IU bolus followed by 1250 IU/h for 6 h or 5000 IU bolus followed by 500 IU/h for 6 h), both, or neither during EVT for AIS caused by LVO in the anterior circulation [3]. The results showed that neither aspirin nor heparin improved functional outcomes at 90 days, moreover both were associated with an increased risk of sICH [3]. A retrospective analysis of 619 patients from the Chinese ANGEL registry revealed that heparin (IV administration of unfractionated heparin, being infused at 50–100 IU/Kg at first and additional 1,000 IU at intervals of an hour during the operation) during EVT was associated with higher rates of sICH and distal embolization [13]. In contrast, a retrospective study evaluating 76 patients with AIS due to intracranial internal carotid artery or middle cerebral artery M1 segment occlusion reported lower rates of sICH and higher rates of reperfusion if heparin was used [14]. Possibly, besides the study designs, variations in baseline characteristics among the study cohorts could explain these discrepancies. It is important to emphasiaze that heparin was typically administered as a bolus intra-procedurally.

Sub-studies of the TREVO-2 trial and the MERCI trial revealed that heparin had neutral effects on the occurrence of sICH and mortality, while exhibiting a positive impact on overall outcome [15, 16]. Importantly, TREVO-2 and MERCI trials did not restrict periprocedural heparin use and it was administered at the discretion of the operator [15, 16]. A study on data from the German Stroke registry, evaluating the use of heparin in both anterior and posterior circulation during EVT, found that administering a periprocedural heparin bolus was associated with worse functional outcomes in patients with occlusion in the anterior circulation whereas no effect on functional outcomes and safety was observed in posterior circulation stroke [17].

Multiple studies including trials (IST trial [18], TOAST trial [19]), meta-analyses [20] and large cohort studies [21] reveal higher sICH rates in patients with heparin use in AIS. In a commentary by Hatchinski the potential risk of sICH associated with the widespread use of heparin was highlighted [22]. In one review, the authors concluded that patients with AIS who have symptomatic large artery stenosis > 70% or who have a non-occlusive intraluminal thrombus may benefit from heparin treatment [23].

This ambiguity in different studies might be theoretically attributed to variations in heparin administration methods such as IV bolus, flush fluids, or a combination of both. Moreover, in some studies the specific way and amount of heparin administration, was not explicitly stated [15, 16].

An often neglected proportion of heparin is administered through flush fluids.

Observations from a Dutch national survey on use of heparin in flush fluids revealed that use of heparin administration during EVT was not standardized, leading to wide variations in how heparin was used, with differences in dose, timing, and method of administration This could potentially lead to high total heparin amounts. One study has focused on the impact of heparinized flush (both the saline fluids and its consequences and the heparin within those fluids), in neurovascular non-stroke procedures [24]. In this study, the fluids administered by both the treating physician and the anesthesiologist were carefully monitored. The median dose of heparin administered was 2500 IU ± 1200 IU per procedure. They concluded that heparin administered with the saline flush had a strong anticoagulant effect, as evidenced by a prolonged activated partial thromboplastin time.

Our international survey of neurointerventionalists revealed a variety in heparin doses administered through both flush fluids and IV bolus in Europe. The median flow rate was 540 IU/h (IQR 180–1080) with a maximum of 9000 IU/h. This was lower than in the Dutch survey which showed a median flow rate of 1350 IU/h (IQR of 2700 IU/h) with a maximum of 13,500 IU/h [2]. This was mainly due to differences in heparin flush concentrations used, ranging from no heparin to 25,000 IU/L [2].

The observed variation in heparin use during EVT, both in flush fluids and as IV bolus, mirrors the variability seen among treating physicians in and outside of Europe. The wide variation reflects the lack of standardized protocols for heparin use. Notably, the administration of high heparin doses during long and complex EVT procedures raises concerns. Given the recent literature on the risks of heparin, we caution physicians to be aware of the "unknown" amount of heparin administered through flush fluids and recommend limiting its use. Measuring Activated Clotting Time (ACT) during EVT would be essential for future studies on use of heparin during EVT. Currently, an optimal ACT level for EVT in clinical practice is not known. However, maintaining an appropriate ACT range could potentially ensure that anticoagulation is effective in preventing thrombus formation without increasing the risk of haemorrhage.

Specifically, for the flush fluid heparin administration, doses are often unknown since most variables are not routinely documented. Therefore, we advise monitoring heparin doses in future randomized trials in order to include this variable as a potential confounder and to provide insight in optimal dose of heparin.

### Limitations

Inherent to all surveys, not all participants responded questions comprehensively. To accurately calculate the total heparin dose, it was crucial for all respondents to answer all questions comprehensively. However, some respondents did not complete all questions, perhaps not all variables were documented, reducing the number of usable responses and most likely underestimating the variability currently shown. Also, this survey represents the standard procedure settings and we assume that there is within centers an important variation regarding the heparin dose since the duration of thrombectomies is highly variable from case to case. Additionally, the limitation of the methodology is that the problem has not been addressed with sufficient precision. Specifically, the calculation of the amount of heparin administered by the proceduralist during the procedure is imprecise due to various confounding factors. These include the duration for which a flush is kept open or attached to the catheter, as well as the variability in the speed of the flush, which is not always steady during the procedure. Nonetheless, the main message of this survey was to provide sufficient data to calculate the total heparin flush dose, including detailed variables, and to show variety of heparin doses administered through flush fluids. We were able to do that for over 100 neurointerventionalists from various parts of the world.

## Conclusions

Use of heparin during EVT varies widely among physicians worldwide. The amount of heparin administered through flush fluids is mostly unknown but can reach high levels if not paying attention. Neurointerventionalists who perform EVT for AIS should be aware of this, especially when using high drip rates, multiple infusion bags, and during long procedures. Studying optimal heparin levels in flush fluids would be needed in future research, while heparin flush doses should be considered in trials regarding periprocedural anticoagulants. This survey underlines the need for guidelines or recommendations on heparin use, either as bolus or in flush fluids, during EVT for AIS.

## Supplementary Information

Supplementary Material 1.

Supplementary Material 2.

## Data Availability

No data are available. Not applicable.
