# Supplementary material for: The variable use of heparin through intravenous bolus and flush fluid systems during endovascular stroke treatment, a world-wide survey
Source: CVIR Endovasc. 2025 Mar 3;8:17. doi: 10.1186/s42155-025-00532-3 (PMC11872961; doi:10.1186/s42155-025-00532-3)
Supplement: Supplementary file 1 — Supplementary Material 1. [file 42155_2025_532_MOESM1_ESM.pdf]

Supplemental

Table S1. Calculated total heparin dose per hour through flush fluids

	Respondents (N=139)
Heparin dose per hour (IU/h) – Median [IQR]	540 [180 - 1080]
Heparin dose per hour <1000 (IU/h) – n (%)	100 (71,9)
Heparin dose per hour 1000-2000 (IU/h) – n (%)	23 (16,5)
Heparin dose per hour 2000-2300 (IU/h) – n (%)	3 (2,2)
Heparin dose per hour 2300-3000 (IU/h) – n (%)	6 (4,3)
Heparin dose per hour >3000 (IU/h) – n (%)	7 (5,0)

Table S2. Calculated total heparin dose per hour for administered IV bolus

	Respondents (N=23)
Total heparin dose per hour (IU/h) – Median [IQR]	4650 [3432 - 5900]
Total heparin dose per hour <1000 (IU/h) – n (%)	2 (8,6)
Total heparin dose per hour 1000-2000 (IU/h) – n (%)	1 (4,3)
Total heparin dose per hour 2000-2300 (IU/h) – n (%)	1 (4,3)
Heparin dose per hour 2300-3000 (IU/h) – n (%)	1 (4,3)
Total heparin dose per hour >3000 (IU/h) – n (%)	18 (78,2)
