# Supplementary material for: The variable use of heparin through intravenous bolus and flush fluid systems during endovascular stroke treatment, a world-wide survey
Source: CVIR Endovasc. 2025 Mar 3;8:17. doi: 10.1186/s42155-025-00532-3 (PMC11872961; doi:10.1186/s42155-025-00532-3)
Supplement: Supplementary file 2 — Supplementary Material 2. [file 42155_2025_532_MOESM2_ESM.pdf]

Heparin use in stroke thrombectomy

Heparin use in stroke thrombectomy: A European Survey

This survey is designed to obtain an overview of the use of any heparin in endovascular thrombectomy practices in Europe. It is supported by the ESMINT society and EYMINT research collaborative group.

Heparin during endovascular thrombectomy (EVT) for acute ischemic stroke (AIS) and its impact on functional outcome is often underestimated, which is reflected by the results of the MR CLEAN MED trial(1). The use of heparin in infusion bags, that flushes the catheters during the treatment, is not considered in the recent trials regarding the impact of antithrombotics during EVT(1,2) and could have influenced the results.

However, the heparin dose during EVT (i.e., as IV bolus and/or as heparin in flush fluids infusion bags) has high inter-individual variability, mainly due to procedural factors (e.g., whether or not an IV bolus is given, the number of infusion bags used, the timing of connecting the bag, drip-rate). In order to truly analyze the impact on functional outcome after EVT, this variability should first be investigated.

This survey will provide more insight in how European interventionists consider their heparin administration during EVT. Data will be treated anonymously and will be used for further research projects regarding heparin (flush) use in EVT for AIS and its impact on outcome.

References:

1. Van der Steen W, van de Graaf RA, Chalos V, et al. Safety and efficacy of aspirin, unfractionated heparin, both, or neither during endovascular stroke treatment (MR CLEAN-MED): an open-label, multicentre, randomised controlled trial. The Lancet. 2022;399(10329):1059-1069. doi:10.1016/s0140-6736(22)00014-9
2. LeCouffe NE, Kappelhof M, Treurniet KM, et al. A Randomized Trial of Intravenous Alteplase before Endovascular Treatment for Stroke. N Engl J Med. Nov 11 2021;385(20):1833-1844. doi:10.1056/NEJMoa2107727

Heparin use in stroke thrombectomy

* 1. In which center are you mainly performing EVT ?

* 2. Are you a :

- ◯ Radiologist
- ◯ Neurologist
- ◯ Neurosurgeon
- ◯ Other (please specify)

* 3. Are you a :

- ◯ Resident
- ◯ Fellow
- ◯ Medical specialist / consultant
- ◯ Other (please specify)

* 4. Gender (only one answer)

- ◯ Female
- ◯ Male

* 5. How many years of experience do you have in performing EVT?

◯ < 1 year

◯ 1-5 years

◯ 5-10 years

◯ > 10 years

* 6. Do you give heparin during EVT in any way (e.g., as a fixed IV bolus, in flush fluids)?

◯ Yes

◯ No

Heparin use in stroke thrombectomy

* 7. For what access route do do you preferably give heparin?

- [ ] Radial
- [ ] Humeral / brachial
- [ ] Femoral
- [ ] Carotid
- [ ] All of the above

* 8. How do you administer heparin?

- ◯ IV bolus
- ◯ Via infusion bags in flush fluids
- ◯ Both

Heparin use in stroke thrombectomy

IV-bolus

This page includes questions about the administration of an IV heparin bolus.

* 9. For IV bolus: What is the dose? This should be a standardized number input (IU/kg)

* 10. For IV bolus: When do you give this dose during the procedure ?

- ◯ Right after arterial puncture
- ◯ At the moment when the thrombectomy device reaches the thrombus
- ◯ After thrombectomy

Heparin use in stroke thrombectomy

Flush fluids.

This page includes questions regarding the administration of heparin in flush fluids.

* 11. For flush fluids: What is the dose per 1L NaCL (please re-calculate if you use 500 mL NaCL)? This should be a standardized number input (i.e., IU/L)

* 12. For flush fluids: How many infusion bags do you simultaneously use to flush catheters during EVT?

◯ 1

◯ 2

◯ 3

◯ > 3

* 13. For flush fluids: How do you manage the drip rate?

◯ Manually : I just look at the drip chamber of the infusion bags and adjust where needed

◯ Automatically : I use a perfusor and set this at a fixed rate

ESMINT European Society of Minimally Invasive Neurological Therapy

EYMINT Young Interventionalists @ ESMINT

Heparin use in stroke thrombectomy

Flush fluids

This page includes further questions regarding heparin use in flush fluids.

* 14. Which video corresponds best to your preferred drip rate?

◯ 0,5 drip/sec (90mL/h)

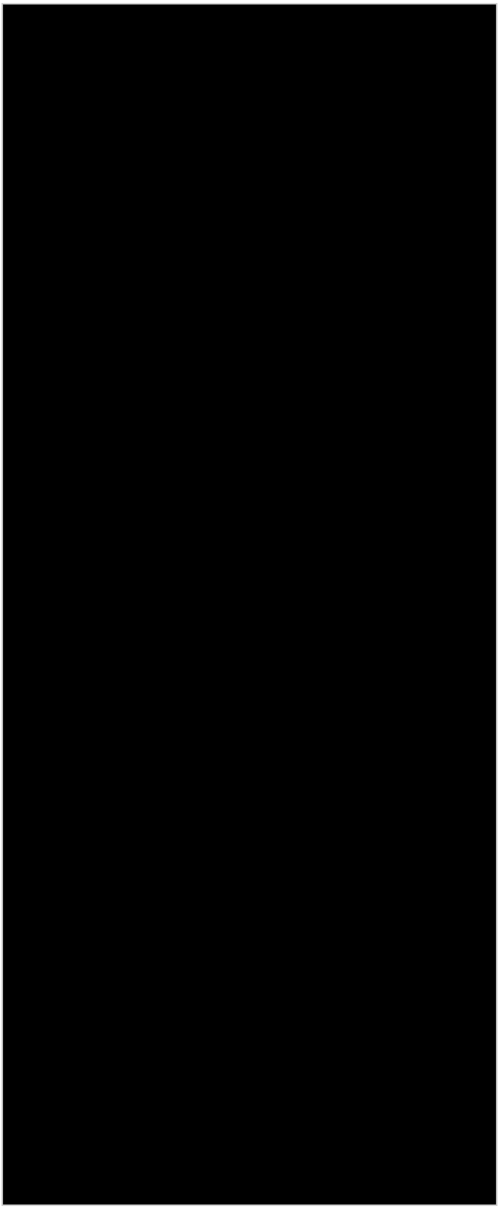

◯ 5 drips/sec (900mL/h)

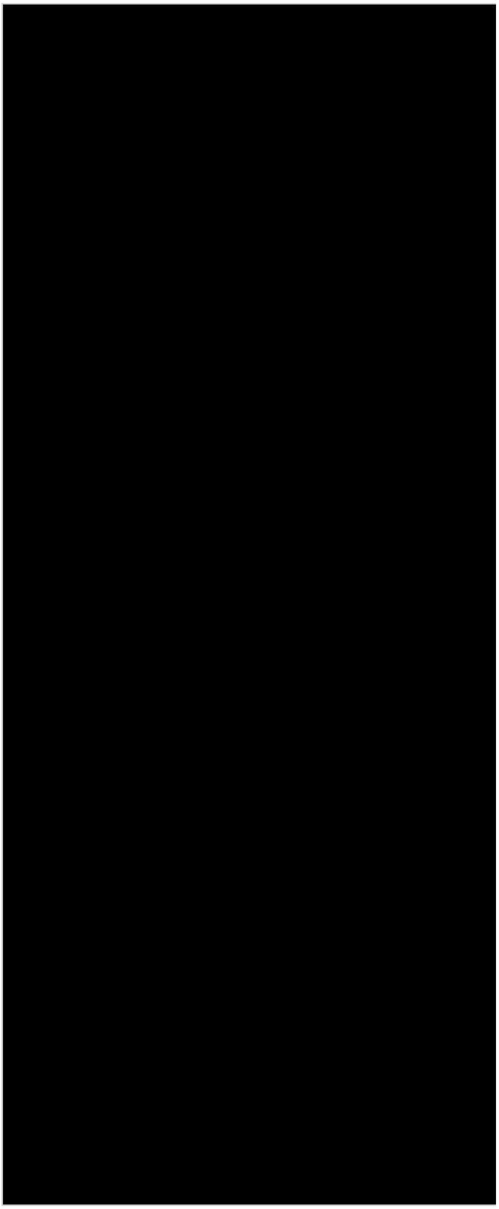

◯ 1 drip/sec (180mL/h)

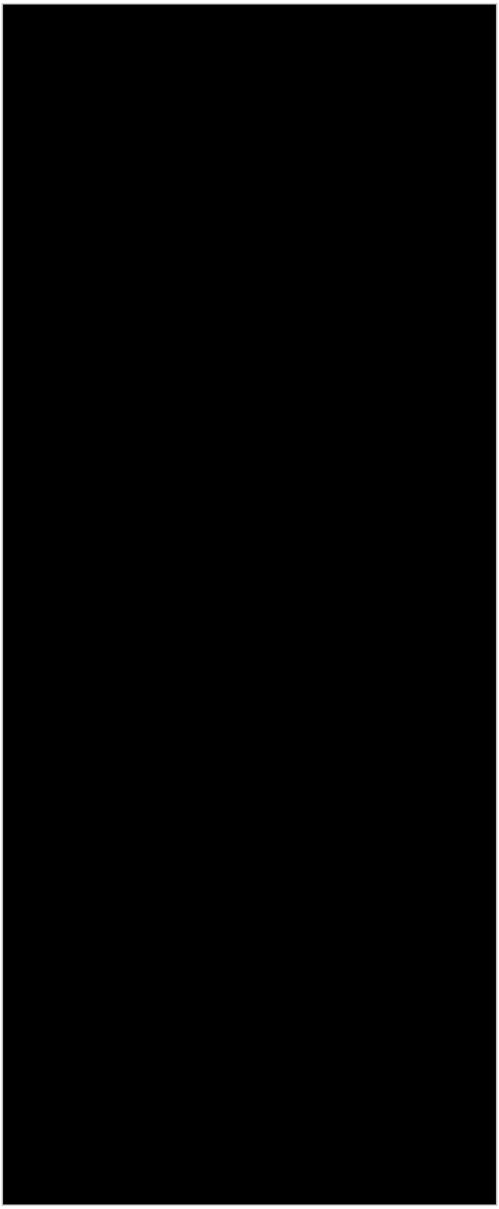

◯ 1 drip/ 5 sec (36mL/h)

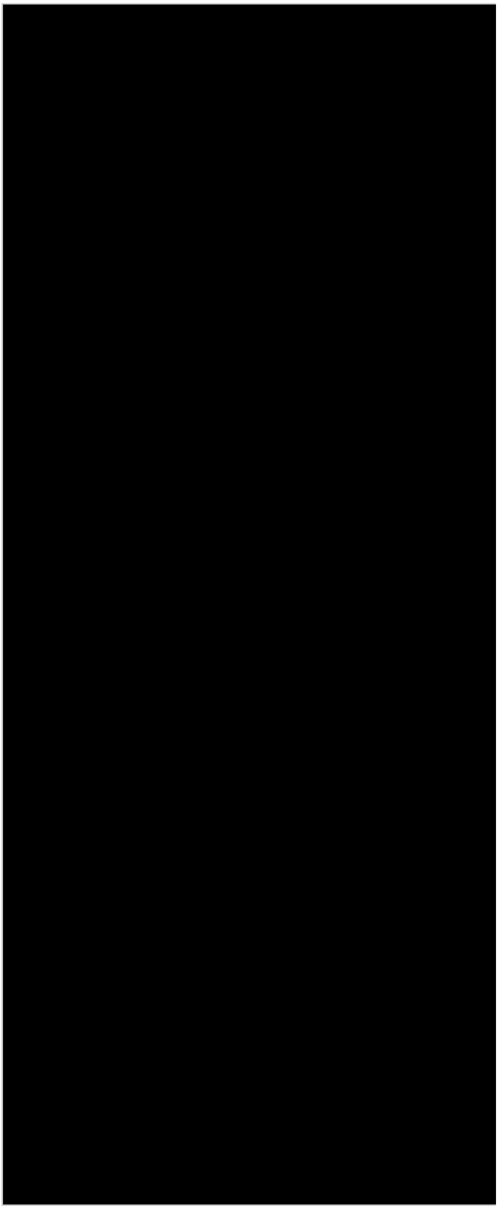

◯ According to the heart rate

◯ 2 drips/sec (360mL/h)

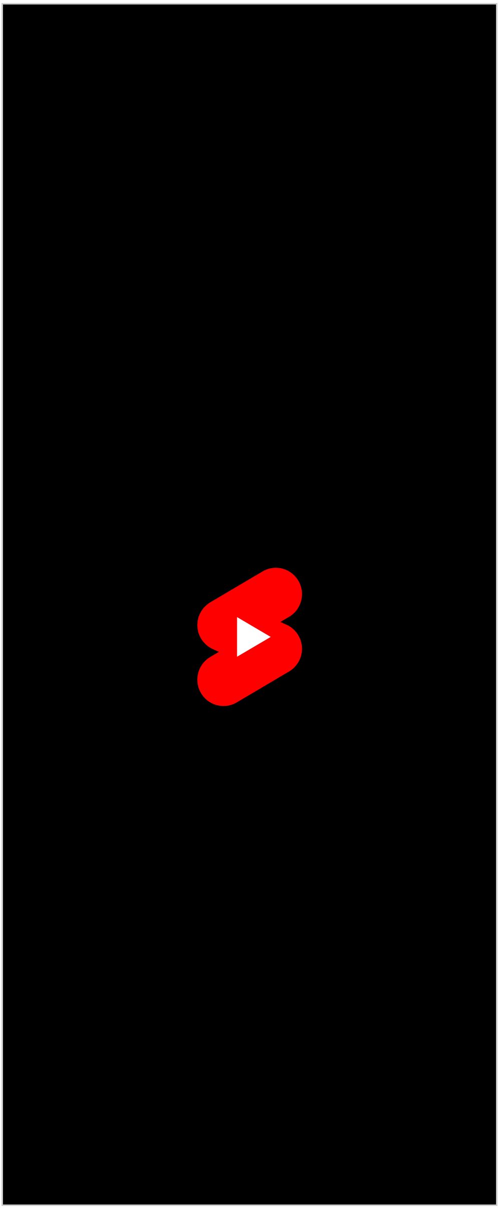

Heparin use in stroke thrombectomy

* 15. What drip rate is this (mL/min)? Please re-calculate if you have a different perfusor unit, other than mL/min. This should be a standardized number input (i.e., mL/min).

Heparin use in stroke thrombectomy

Flush fluids

This page includes further questions regarding heparin use in flush fluids

* 16. When do you connect the infusion bag(s)?

◯ At the start of the procedure, right after or before groin puncture

◯ Only when catheterizing the carotid artery

* 17. Is your speed similar for each bag?

☐ Yes

☐ No, please explain

Heparin use in stroke thrombectomy

IV bolus + flush fluids

This page includes further questions about the administration of heparin in flush fluids and through an IV bolus

* 18. For IV bolus: What is the dose? This should be a standardized number input (IU/kg)

* 19. For IV bolus: When do you give this dose during the procedure ?

- ◯ Right after arterial puncture
- ◯ At the moment when the thrombectomy device reaches the thrombus
- ◯ After thrombectomy

* 20. For flush fluids: What is the dose per 1L NaCL (please re-calculate if you use 500 mL NaCL)? This should be a standardized number input (i.e., IU/L)

* 21. For flush fluids: How many infusion bags do you use simultaneously to flush catheters during EVT?

- ◯ 1
- ◯ 2
- ◯ 3
- ◯ > 3

* 22. For flush fluids: How do you manage the drip rate?

- ◯ Manually : I just look at the drip chamber of the infusion bags and adjust where needed
- ◯ Automatically : I use a perfusor and set this at a fixed rate

Heparin use in stroke thrombectomy

IV bolus + flush fluids

This page includes further questions about the administration of heparin in flush fluids and through an IV bolus

* 23. Which video corresponds best to your preferred drip rate?

◯ 0,5 drip/sec (90mL/h)

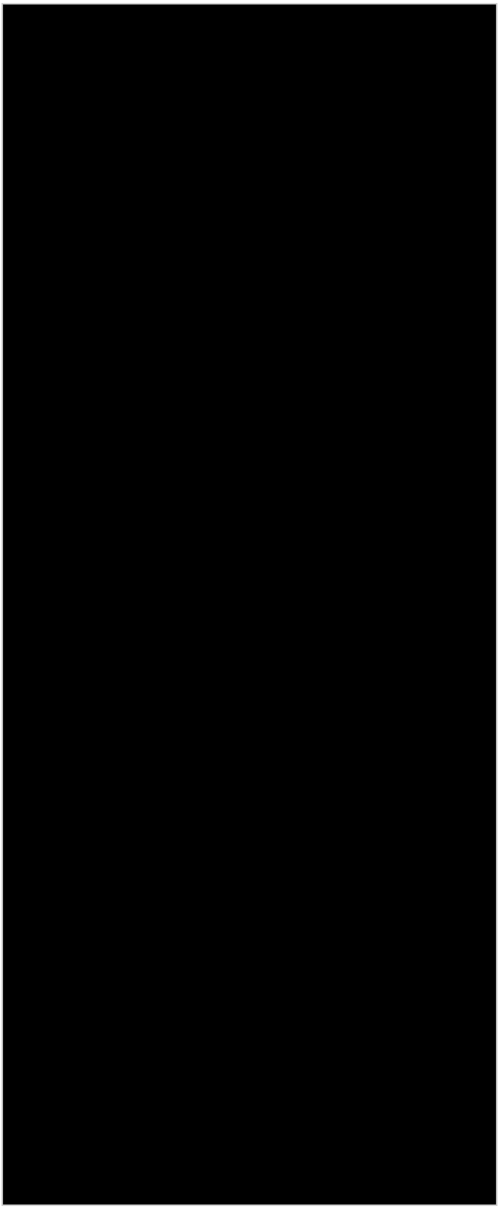

◯ 5 drips/sec (900mL/h)

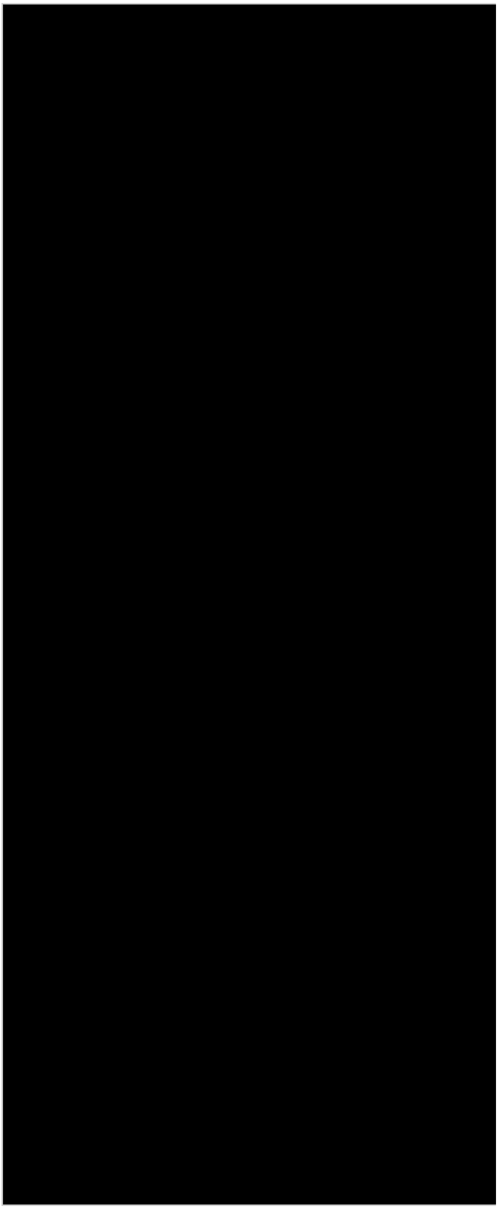

◯ 1 drip/sec (180mL/h)

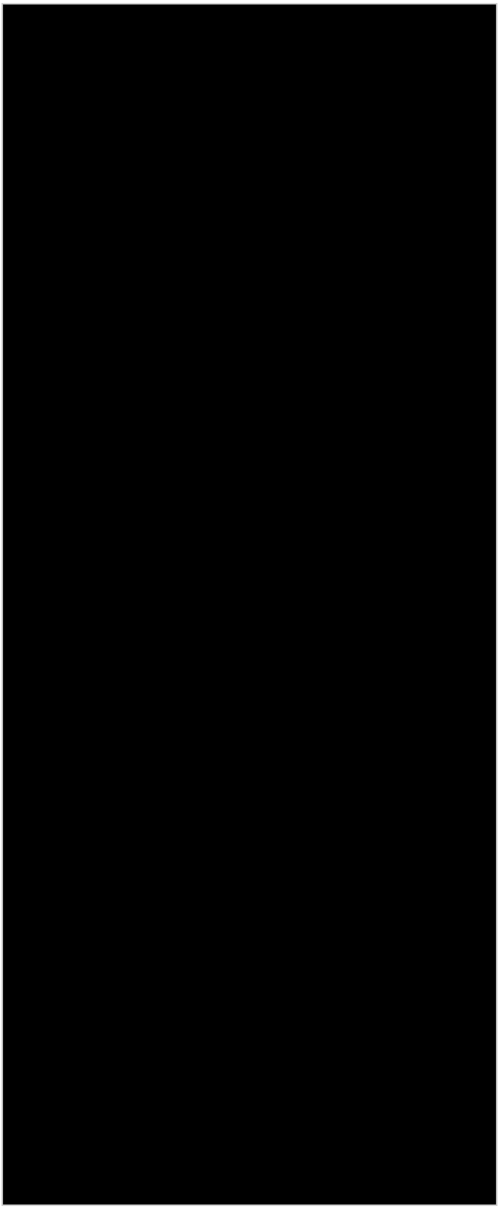

◯ 1 drip/ 5 sec (36mL/h)

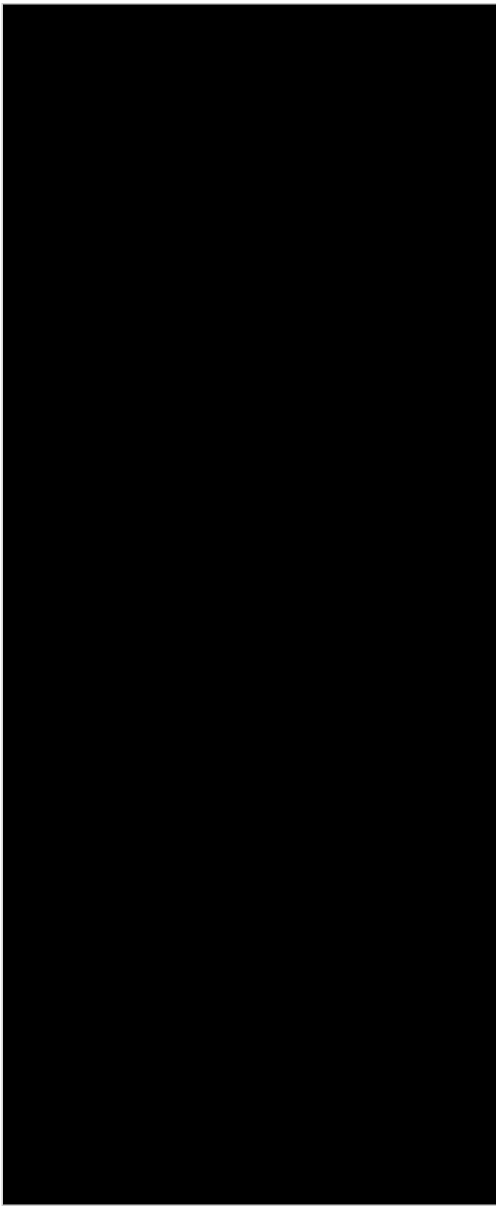

◯ According to the heart rate

◯ 2 drips/sec (360mL/h)

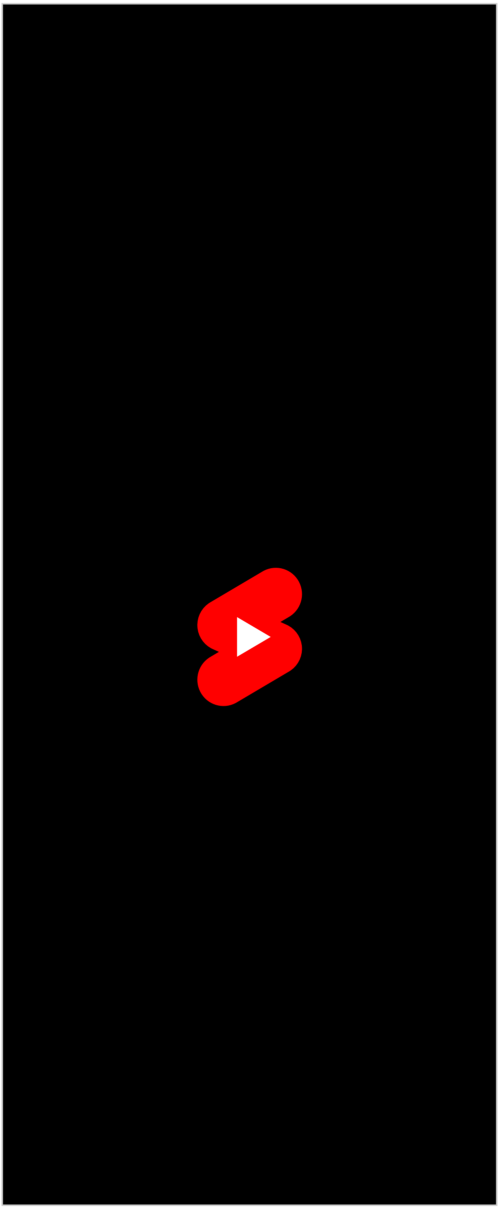

* 24. When do you connect the infusion bag(s)?

◯ At the start of the procedure, right after or before groin puncture

◯ Only when catheterizing the carotid artery

25. Is your speed similar for each bag?

☐ Yes

☐ No, please explain:

Heparin use in stroke thrombectomy

* 26. What drip rate do you use (mL/min)? Please re-calculate if you have a different perfusor unit, other than mL/min. This should be a standardized number input (i.e., mL/min).

Heparin use in stroke thrombectomy

* 27. Who manages this heparin administration?

- ◯ The interventionist
- ◯ The x-ray technician
- ◯ The anesthesiologist
- ◯ The anesthesia technician
- ◯ Other (please specify)

* 28. Do you withhold heparin administration in certain cases?

- ◯ Yes
- ◯ No

Heparin use in stroke thrombectomy

* 29. What are these cases (you can mark ≥1 answer)?

- [ ] When IV thrombolysis is given
- [ ] If patient has low ASPECTS (<5)
- [ ] In older patients (>80 years)
- [ ] In patients with extensive brain atrophy and/or features of small vessel disease, detected on NCCT / MRI
- [ ] When I am planning to stent the carotid artery and/or intracranially
- [ ] When reperfusion was unsuccessful (only for heparin bolus)
- [ ] Other (please specify)

We thank you for your time and please don't forget to submit.

Sincerely,

ESMINT & EYMINT on behalf of Faysal Benali and Wim van Zwam
